# Dietary Patterns and Their Associations with Intermediate Age-Related Macular Degeneration in a Japanese Population

**DOI:** 10.3390/jcm11061617

**Published:** 2022-03-15

**Authors:** Mariko Sasaki, Naoko Miyagawa, Sei Harada, Kazuo Tsubota, Toru Takebayashi, Yuji Nishiwaki, Ryo Kawasaki

**Affiliations:** 1 Department of Ophthalmology, Keio University School of Medicine, Tokyo 160-8582, Japan; tsubota@z3.keio.jp; 2 Department of Ophthalmology, Tachikawa Hospital, Tokyo 190-8531, Japan; 3 National Institute of Sensory Organs, National Tokyo Medical Center, Tokyo 152-8902, Japan; 4 Department of Preventive Medicine and Public Health, Keio University School of Medicine, Tokyo 160-8582, Japan; nmiya@keio.jp (N.M.); seipeace2u@gmail.com (S.H.); ttakebayashi@keio.jp (T.T.); 5 Department of Environmental and Occupational Health, Toho University, Tokyo 143-8540, Japan; yuuji.nishiwaki@med.toho-u.ac.jp; 6 Department of Vision Informatics (Topcon), Osaka University Graduate School of Medicine, Osaka 565-0871, Japan; ryo.kawasaki@ophthal.med.osaka-u.ac.jp

**Keywords:** age-related macular degeneration (AMD), intermediate AMD, dietary pattern, principal component analysis, Asian, staple food

## Abstract

This population-based cross-sectional study investigated the influence of dietary patterns on age-related macular degeneration (AMD) in a Japanese population. The Tsuruoka Metabolomics Cohort Study enrolled a general population aged 35–74 years from among participants in annual health check-up programs in Tsuruoka City, Japan. Eating habits were assessed using a food frequency questionnaire. Principal component analysis was used to identify dietary patterns among food items. The association between quartiles of scores for each dietary pattern and intermediate AMD was assessed using multivariate logistic regression models. Of 3433 participants, 415 had intermediate AMD. We identified four principal components comprising the Vegetable-rich pattern, Varied staple food pattern, Animal-rich pattern, and Seafood-rich pattern. After adjusting for potential confounders, higher Varied staple food diet scores were associated with a lower prevalence of intermediate AMD (fourth vs. first quartile) (OR, 0.63; 95% confidence interval [CI], 0.46–0.86). A significant trend of decreasing ORs for intermediate AMD associated with increasing Varied staple food diet scores was noted (*p* for trend = 0.002). There was no significant association between the other dietary patterns and intermediate AMD. In a Japanese population, individuals with a dietary pattern score high in the Varied staple food pattern had a lower prevalence of intermediate AMD.

## 1. Introduction

Age-related macular degeneration (AMD) is a leading cause of visual loss among older people worldwide [1], including in Japanese and other Asian populations [2]. Asians currently comprise 60% of the world’s population and are projected to account for largest share of the global prevalence of AMD by 2040 [3]; thus, AMD in Asia is becoming an increasingly important public health problem globally. 

A number of studies have demonstrated the associations between dietary intake (including lutein and zeaxanthin [4,5,6,7], omega 3 polyunsaturated fatty acids, and fish [8,9,10,11]) and reduced risk of AMD. Most of these studies investigated the associations of AMD with a single nutrient or food item; however, a diet is a combination of several nutrients and foods that may have synergetic effects on AMD pathology [12]. Therefore, it is also important to assess the association between AMD risk and dietary patterns. Several studies have investigated the associations between AMD risks and predetermined dietary patterns [13,14,15,16]. The Mediterranean diet, which is characteristically high in plant foods and olive oil, low-to-moderate in fish, dairy products, and wine, and low in meat [17], has been associated with a reduced risk of AMD [14,18,19]. In contrast, the Western diet characterized by high intakes of red meat, processed meat, high-fat dairy products, refined grains, and high alcohol consumption, has been associated with an increased risk of AMD [19,20,21]. These findings suggest that a healthy nutrient-rich diet could contribute to the prevention of AMD. 

Principal component analysis (PCA) simplifies high-dimensional data to capture trends and patterns, and it can identify patterns without reference to prior knowledge [22]. It has recently been used in epidemiological studies, but to date only a few studies have used it to assess the association between dietary patterns and AMD risk and only in Western populations [20,23].

Because dietary patterns are diverse and indigenous to the lesion and ethnicity, and differ between Western and Asian populations, the influence of dietary patterns on AMD risk may differ between these populations. We therefore aimed to examine the cross-sectional associations of dietary patterns with AMD in a Japanese cohort from the Tsuruoka Metabolomics Cohort Study.

## 2. Methods

### 2.1. Study Participants

This study was based on data derived from participants in the Tsuruoka Metabolomics Cohort Study [24,25]. In brief, from April 2012 to March 2015, 71,868 residents with national health insurance coverage in Tsuruoka City, Japan, aged 35–74 years, were identified, and 11,001 residents were enrolled from among those who took part in annual municipal health check-up programs that included fundus photographs in the city. Of these, 8415 participants completed a food frequency questionnaire (participation rate, 11.7%). The present study, which is a sub-study of the overall Tsuruoka Metabolomics Cohort Study, included a total of 4010 individuals who participated in the baseline survey between April 2012 and March 2013.

This study was conducted in accordance with the Ethical Guidelines for Medical and Health Research Involving Human Subjects, Japan, and was approved by the Medical Ethics Committees of the School of Medicine, Keio University, Tokyo, Japan (Approval No. 20110264) and the School of Medicine, Toho University, Tokyo, Japan (Approval No. 26028). Written informed consent was obtained from all participants included in the study.

### 2.2. Data and Sample Collection

Each participant underwent a comprehensive assessment including a range of clinical, biochemical, and anthropometric measurements. Lifestyle factors were assessed using validated questionnaires [26]. All data and samples were obtained during annual health checkups.

Non-stereoscopic fundus photographs of one eye (generally the right eye) were obtained using a 45° non-mydriatic fundus camera (TRC-NW200; Topcon Corp., Tokyo, Japan) without use of pharmacological dilating agents. Images were centered on the optic disc and macula. If fundus photography of the right eye was not possible because of media opacity or other causes, the left eye was then photographed (n = 126).

Blood pressure (BP) was measured twice after participants had been seated comfortably for at least 5 min, using an automated sphygmomanometer (Omron HBP-T105S-N). The means of two measurements of systolic and diastolic BP were used for analysis. Body mass index was calculated as weight (kg) divided by height squared (m^2^). 

Blood samples were collected between 8:30 a.m. and 10:30 a.m. after overnight fasting to avoid variations due to fasting state and circadian rhythm. Serum levels of total cholesterol, triglyceride (TG), and fasting plasma glucose were analyzed using enzymatic methods, and glycated hemoglobin (HbA1c) was determined by immunoassay. High-density lipoprotein cholesterol (HDL-C) values were measured by a direct method. Serum lipid levels were confirmed by the Japan Medical Association for precision and validity for standardized testing [27]. Low-density lipoprotein cholesterol (LDL-C) levels were calculated using the Friedewald formula at plasma TG levels ≤400 mg/dL. 

Information regarding smoking history, alcohol intake, and use of antihypertensive, antidiabetic, and lipid-lowering medications was obtained using a standardized self-administered questionnaire. Information on CVD history was obtained by surveying the history of all visits to the main hospital in the region. Hypertension was defined as systolic BP ≥ 140 mmHg, diastolic BP ≥ 90 mmHg [28], or use of antihypertensive medication. Diabetes was defined as fasting plasma glucose ≥ 126 mg/dL or HbA1c (National Glycohemoglobin Standardization Program) ≥ 6.5% or use of antidiabetic medication. Dyslipidemia was defined as LDL-C ≥ 140 mg/dL, HDL-C < 40 mg/dL, TG ≥ 150 mg/dL, or use of lipid-lowering medication. Obesity was defined as body mass index ≥25 kg/m^2^.

### 2.3. Dietary Patterns

Dietary intake was assessed using a previously validated short food frequency questionnaire [29,30,31]. Dietary data included the frequency of habitual dietary intake during the previous year for 47 food items.

To identify dietary patterns, PCA was performed based on intake of these 46 food items (excluding alcohol consumption) [23,32]. The frequency of foods/dishes was standardized before entry into PCA. The number of major dietary patterns was determined from scree plots, eigenvalues (>1.5), and the interpretability of each component in the PCA results. The greater the magnitude of positive or negative factor loadings, the more important the food is to that component. Foods with factor loadings of 0.2 or higher were interpreted as contributing significantly to the dietary patterns.

### 2.4. Grading of Fundus Photographs

A non-mydriatic fundus photograph of one eye of each participant was evaluated to determine whether the quality was sufficient for grading AMD lesions. Fundus photographs were evaluated at the Yamagata University reading center (principal investigators: RK and YK). AMD photographs were graded according to the protocol used in the Blue Mountain Eye Study (BMES) [33] and were classified based on the modified Age-Related Eye Disease Study (AREDS) classification [34]. In brief, a trained grader (YK) assessed the photographs for signs of AMD in a masked fashion, and in consultation with a senior researcher (RK) when grading uncertainties were noted.

The severity of AMD was defined as follows: Category 1 (normal), two or fewer hard drusen (diameter < 63 μm); Category 2 (early AMD), three or more hard drusen and/or two or fewer soft drusen (diameter ≥ 63 but < 125 μm) and/or a retinal pigment epithelium abnormality; Category 3 (intermediate AMD), any large drusen (diameter ≥ 125 μm) and/or three or more soft drusen and/or geographic atrophy within the grid but none at the center of the macula; and Category 4 (late AMD), neovascular AMD and/or geographic atrophy in the central subfield. Neovascular AMD was defined as the presence of fibrovascular/serous pigment epithelial detachment, serous (or hemorrhagic) sensory retinal detachment, subretinal/subretinal pigment epithelial hemorrhage, subretinal fibrous tissue (or fibrin), or photocoagulation for AMD. Geographic atrophy was defined as a sharply demarcated, usually circular, zone of partial or complete depigmentation of the retinal pigment epithelium, typically with exposure of the underlying large choroidal blood vessels. We defined AMD as Category 3 or higher in this study.

### 2.5. Statistical Analysis

After dietary patterns were identified by PCA as described above, participants were divided into quartiles by scores for each dietary pattern. Baseline participant characteristics were then summarized according to the dietary patterns.

The association between quartiles of score for each dietary pattern and AMD was assessed using 2 multivariate logistic regression models with the lowest quartile as the reference group and expressed as odds ratios (ORs) with 95% confidence intervals (CIs). The first model was adjusted for age and sex. The second model was adjusted for age, sex, smoking status, alcohol consumption, presence of hypertension, diabetes or dyslipidemia, and history of cardiovascular disease. Analysis items with a two-tailed *P*-value of less than 0.05 were considered statistically significant. All statistical analyses were performed using SAS version 9.4 for Windows (SAS Institute Inc., Cary, NC, USA).

## 3. Results

A total of 333 men and women were excluded from this analysis for the following reasons: 22 with missing fundus images or suboptimal fundus image quality (i.e., poor focus, eyelash artifacts, or uneven illumination) and 316 with missing information from the dietary survey (n = 304) or for other covariates (n = 12) (Includes multiple reasons). Excluded participants were older and had higher levels of fasting plasma glucose and HbA1c and had higher frequencies of diabetes, current smokers and current drinkers. In addition, we excluded subjects with Category 2 AMD (n = 244) because we defined AMD as Category 3 or higher in this study. Although participants with Category 2 AMD were excluded, we note that there was no significant association between dietary patterns and the prevalence of participants with Category 2 (Supplemental Table S1). There were no significant differences in demographics between the final study population and the overall population.

Ultimately, we included the remaining 3433 participants (1561 men and 1872 women) in the present cross-sectional analysis. There were 415 participants with Category 3 AMD, and none with Category 4. Basic characteristics according to AMD status are presented in Table 1. Participants with intermediate AMD were older and had higher levels of systolic BP and HDL-C and lower levels of TG and had higher frequencies of hypertension and a history of CVD. Four factors were identified by PCA. Factor loadings are shown in Table 2. Based on the interpretability of the factors, the four factors were designated as follows: (1) Vegetable-rich dietary pattern, characterized by high intake of vegetables, potatoes, seaweed, and mushrooms; (2) Varied staple food dietary pattern, characterized by high intake of bread and noodles, margarine on bread, and yogurt and low intake of rice and miso soup; (3) Animal-rich dietary pattern, characterized by high intake of meat (including chicken, beef/pork, and processed meat), eggs, shellfish, and fish eggs, deep fried food, mayonnaise, and Western confectionery; and (4) Seafood-rich dietary pattern, characterized by high intake of seafood (including bone-edible small fish, shellfish, and fish eggs) and low intake of meat, pan-fried food, and coffee. The frequency of intake of foods/dishes per week in each dietary pattern, divided into quartiles for each dietary pattern score, is shown in Supplemental Table S2. Furthermore, the weekly frequency of staple food intake at each meal of the day for each dietary pattern is shown in Supplemental Table S3. Participants with higher Varied staple food scores were likely to eat bread or noodles instead of rice for breakfast or lunch. 

Basic characteristics according to quartile of dietary pattern score are presented in Supplemental Table S4. Participants with higher scores in the Vegetable-rich pattern were older and more likely to be women, to be a non-smoker, have lower alcohol consumption, and have lower levels of fasting plasma glucose, triglycerides, and LDL-C than those with lower scores. Participants with higher scores in the Varied staple food pattern were younger and more likely to be women, have lower alcohol consumption, have lower body mass index and systolic and diastolic BP, but have higher levels of total cholesterol and LDL-C than those with lower scores. For the Animal-rich pattern, participants with higher scores were younger and more likely to be men and a current smoker, less likely to have hypertension or dyslipidemia, and have higher alcohol consumption. For the Seafood-rich pattern, participants with higher scores were older and more likely to be men and a current smoker, to have diabetes, have higher BP, have higher levels of plasma fasting glucose and triglycerides, and have higher alcohol consumption than those with lower scores. 

After adjusting for age, sex, smoking status, alcohol consumption, presence of hypertension, diabetes, or dyslipidemia, higher scores in the Varied staple food pattern were associated with a lower prevalence of intermediate AMD (highest vs. lowest quartile) (OR, 0.63; 95% confidence interval [CI], 0.46–0.86) (Table 3). A significant trend of decreasing ORs for intermediate AMD associated with increasing Varied staple food diet scores was noted (*p* for trend = 0.002). There was no significant association between the other dietary patterns and intermediate AMD. Additionally, 13 out of 310 participants aged between 39 to 50 years had intermediate AMD. However, with the exclusion of these 310 participants, the results did not change markedly (Supplemental Table S5). No significant interaction was found between any dietary patterns and intermediate AMD.

## 4. Discussion

We analyzed dietary patterns in a community-dwelling Japanese population with 3433 participants by using PCA and identified four major dietary patterns. Among these patterns, the Varied staple food pattern characterized by high consumption of bread and noodles but low consumption of rice was associated with a lower prevalence of intermediate AMD. A significant trend of decreasing risk for intermediate AMD associated with increasing scores was noted.

In the Melbourne Collaborative Cohort Study [23], 2508 participants were characterized by the following six factors obtained by PCA: predominant intake of fruits (Factor 1); vegetables (Factor 2); grains, fish, steamed or boiled chicken, vegetables, and nuts (Factor 3); red meat (Factor 4); processed foods comprising cakes, sweet biscuits and desserts (Factor 5); and salad (Factor 6). That study found that higher Factor 3 scores and lower Factor 4 scores were associated with a lower prevalence of advanced AMD. In the Age-Related Eye Disease Study [20], two dietary patterns were identified. The Oriental pattern was characterized by high consumption of vegetables, legumes, fruits, whole grains, tomatoes, and seafood, whereas the Western pattern was characterized by high consumption of red meat, processed meat, high-fat dairy products, French fries, refined grains, and eggs. The Oriental pattern was found to be associated with reduced risk of early and advanced AMD, whereas the Western pattern was associated with increased risk. These findings were consistent with the results of previous studies that suggested diets rich in fruit, vegetables, fish, and nuts are protective against AMD [19,23,35]. This contrasts with the present findings that the Animal-rich dietary pattern and Seafood-rich diet pattern, which correspond to the Western and Eastern dietary patterns in the previous report, were not associated with intermediate AMD. Japanese people consume less red meat and dairy products and more fish and seafoods compared with Western populations [36]. Lower consumption of red meat and dairy products may reduce the risk of intermediate AMD in those with an Animal-rich dietary pattern. On the other hand, higher consumption of fish and seafoods may cause a threshold effect in those with a Seafood-rich dietary pattern. The associations of dietary patterns with AMD could differ among populations with different dietary patterns or genetic backgrounds.

We found an association between habitual eating of staple foods and intermediate AMD in this study. To our knowledge, no previous studies have reported such an association. Meanwhile, a few studies have reported on the association of AMD risk with glycemic index (GI) [37,38,39]. GI ranks carbohydrate foods based on the blood glucose response after carbohydrate food intake compared with that of glucose [40]. Chiu et al. [37] and Kaushik et al. [39] showed that a high-GI diet was associated with an increased risk of early AMD compared with a low-GI diet. Chiu et al. [38] also showed that risk of AMD progression was significantly higher with a high-GI diet than with a low-GI diet. Therefore, a low-GI diet can be beneficial for preventing AMD, and consuming smaller amounts of refined carbohydrates should be recommended [41]. Rice accounts for the majority of cereal grains consumed by Japanese people, and more often than not, it is refined. In the present study, the risk of intermediate AMD was lower among those who ate bread and noodles rather than rice for breakfast and lunch and whose Varied staple food dietary pattern did not rely solely on rice as a staple food. Previous studies on Japanese people reported the GI value of refined rice to be higher than that of bread and noodles, at 69–86, 51–74, and 38–80, respectively [42]. Although calculating GI values was not part of this study’s design, GI might be one of the reasons for the lower risk of intermediate AMD in the group with the higher Varied staple food diet score.

The strengths of this study include its large sample size, use of standardized grading protocols to define AMD by trained graders, and use of validated questionnaires to collect lifestyle information and medical history. We also recognize several limitations of our study. First, because of the cross-sectional design, we cannot draw any conclusions about a causal relationship between the Varied staple food pattern and prevalence of intermediate AMD. Second, because there were no participants with late AMD due to the age range of the study population (35–74 years) or potential healthy screening bias, we were unable to analyze associations with late AMD separately. Third, we used the FFQ for dietary intake estimation, which may have resulted in misclassification. This may have made the association with AMD appear weaker. Fourth, the FFQ used did not have a question to discriminate between refined and unrefined cereals. Fifth, only one non-mydriatic fundus photograph from a single eye was analyzed for each participant. This would underestimate the prevalence of AMD per person. Sixth, the participation rate was relatively low, especially among residents under 60 years old. The age distribution of participants in the present study was not very similar to that of residents in Tsuruoka City overall. Lastly, due to the non-availability of genetic information, drusen secondary to hereditary diseases could not be identified. Therefore, further studies, especially longitudinal studies, will be needed to validate our findings.

In conclusion, we analyzed dietary patterns using PCA in a large Japanese cohort and identified four major dietary patterns. Among these patterns, the Varied staple food pattern was associated with a lower prevalence of intermediate AMD. This dietary pattern does not overemphasize refined white rice as a staple food but also includes other cereals which may be associated partly with the GI value. The effects of dietary patterns on AMD likely differ among different populations due to ethnic and lifestyle variations, and the Varied staple food pattern may reduce the risk of AMD in the Japanese population. Identifying beneficial dietary patterns can help in determining adequate interventional options for preventing or slowing disease incidence or progression.

## Figures and Tables

**Table 1:** Basic characteristics according to age-related macular degeneration (AMD) status.

	AMD Status		*p* Value
	No AMD	Intermediate AMD	
Number	3018	415	
Men, %	45.1	48.2	0.235
Age, years	61.6 (7.8)	65.4 (6.3)	<0.001
Body mass index, kg/m^2^	23.3 (3.3)	23.4 (3.1)	0.401
Systolic BP, mmHg	130.1 (19.4)	132.1 (18.9)	0.042
Diastolic BP, mmHg	76.1 (11.5)	76.5 (11.4)	0.524
Fasting plasma glucose, mg/dL	101.9 (17.4)	101.6 (14.9)	0.684
HemoglobinA1c, %	5.7 (0.6)	5.7 (0.5)	0.451
Total cholesterol, mg/dL	209.9 (33.5)	210.8 (33.3)	0.605
HDL cholesterol, mg/dL	67.8 (17.1)	69.9 (16.2)	0.020
LDL cholesterol, mg/dL	119.7 (30.6)	120.5 (30.9)	0.610
Triglycerides, mg/dL	111.7 (77.8)	101.9 (51.6)	0.001
Hypertension, %	47.2	54.2	0.008
Diabetes, %	11.2	11.8	0.714
Dyslipidemia, %	52.5	53.5	0.690
Obesity, %	27.3	30.1	0.235
History of CVD, %	3.5	6.3	0.006
Smoking status, %			0.401
Never	57.4	58.1	
Past	27.5	29.2	
Current	15.2	12.8	
Current drinker, %	48.0	47.0	0.695
Alcohol consumption, g/day	15.0 (25.9)	16.6 (28.2)	0.263

BP—blood pressure, HDL—high-density lipoprotein, LDL—low-density lipoprotein.

**Table 2:** Factor loadings for 4 principal components derived from habitual dietary patterns.

Food Items	Vegetable Rich	Varied Staple Food	Animal Foods Rich	Seafood Rich
Rice	0.03	−0.48	−0.06	−0.06
Bread	0.01	0.47	−0.01	−0.03
Noodles	0.00	0.27	0.14	0.07
Soba-noodles	0.03	0.19	0.05	0.16
Margarine on bread	0.01	0.33	0.06	−0.06
Butter on bread	0.00	0.15	0.03	0.00
Milk	0.05	0.11	−0.06	−0.05
Yogurt	0.08	0.23	−0.16	0.04
Miso soup	0.06	−0.31	−0.04	−0.01
Tofu products	0.13	−0.04	−0.09	0.17
Soy/Natto (fermented soybeans)	0.12	−0.12	−0.08	0.09
Eggs	0.10	−0.07	0.20	−0.06
Chicken	0.12	0.01	0.22	−0.11
Beef/pork	0.11	−0.04	0.26	−0.25
Liver	0.03	−0.02	0.14	0.10
Processed meat	0.12	0.04	0.27	−0.14
Fish	0.16	−0.10	−0.07	0.16
Bone-edible small fish	0.17	−0.04	−0.06	0.27
Canned tuna	0.10	0.02	0.19	0.07
Octopus/shrimp/crab	0.15	−0.03	0.26	0.29
Shellfish	0.15	−0.01	0.19	0.36
Fish eggs	0.09	−0.09	0.26	0.20
Fish paste products	0.16	−0.07	0.17	0.01
Tofu products	0.19	−0.10	0.01	−0.03
Potatoes	0.22	−0.03	−0.03	0.02
Pumpkin	0.20	0.04	−0.06	0.10
Carrot	0.22	0.02	−0.11	−0.11
Broccoli	0.19	0.05	−0.05	0.07
Green leafy vegetables	0.21	−0.03	−0.21	−0.10
Other green-yellow vegetables	0.21	0.03	−0.16	−0.15
Cabbage	0.22	0.04	−0.15	−0.13
Japanese radish	0.23	−0.03	−0.10	0.02
Dried radish	0.14	0.02	0.02	0.21
Burdock root/bamboo shoot	0.17	0.01	0.02	0.12
Other vegetables	0.21	0.03	−0.12	−0.22
Mushrooms	0.23	0.03	−0.12	−0.16
Seaweed	0.21	−0.03	−0.11	−0.02
Mayonnaise	0.14	−0.01	0.22	−0.18
Deep-fried food	0.13	−0.08	0.31	−0.13
Pan-fried food	0.19	−0.04	0.12	−0.29
Citrus fruits	0.17	0.12	−0.10	0.12
Other fruits	0.19	0.12	−0.16	0.01
Peanuts/almonds	0.11	0.09	0.04	0.14
Western confectionery	0.09	0.10	0.21	−0.04
Japanese confectionery	0.12	0.09	0.06	−0.02
Green tea	0.10	0.00	−0.13	0.07
Coffee	0.04	0.09	0.11	−0.26

**Table 3:** Association of each dietary pattern with the prevalence of intermediate AMD.

	Non-Case/Case	Model 1		Model 2	
Dietary Pattern	n (%)	Odds Ratio (95% CI)	*p* for Trend	Odds Ratio (95% CI)	*p* for Trend
Vegetable rich					
Q1	767/91 (10.6)	Reference		Reference	
Q2	758/100 (11.7)	1.06 (0.78−1.45)	0.827	1.05 (0.77−1.44)	0.791
Q3	746/113 (13.2)	1.14 (0.84−1.56)		1.14 (0.83−1.55)	
Q4	747/111 (12.9)	1.02 (0.74−1.41)		1.03 (0.74−1.42)	
Varied staple food					
Q1	727/131 (15.3)	Reference		Reference	
Q2	749/109 (12.7)	0.86 (0.65−1.13)	0.002	0.85 (0.64−1.13)	0.002
Q3	763/96 (11.2)	0.77 (0.58−1.03)		0.76 (0.57−1.02)	
Q4	779/79 (9.2)	0.63 (0.47−0.86)		0.63 (0.46−0.86)	
Animal foods rich					
Q1	750/108 (12.6)	Reference		Reference	
Q2	736/122 (14.2)	1.29 (0.97−1.71)	0.842	1.28 (0.96−1.70)	0.950
Q3	757/102 (11.9)	1.17 (0.87−1.58)		1.16 (0.86−1.57)	
Q4	775/83 (9.7)	1.06 (0.77−1.46)		1.03 (0.75−1.43)	
Seafood rich					
Q1	777/81 (9.4)	Reference		Reference	
Q2	748/110 (12.8)	1.27 (0.93−1.73)	0.706	1.26 (0.92−1.72)	0.780
Q3	759/100 (11.6)	1.00 (0.73−1.38)		0.99 (0.72−1.37)	
Q4	734/124 (14.5)	1.16 (0.85−1.59)		1.14 (0.84−1.56)	

Model 1, adjusted for age, sex. Model 2, adjusted for age, sex, smoking status, alcohol consumption, presence of hypertension, diabetes, and dyslipidemia, history of cardiovascular disease. AMD—age-related macular degeneration; CI—confidence interval.

## Data Availability

Data available on request due to ethical restrictions. The data presented in this study are available on request from the corresponding author.

## Supplementary Materials

The following are available online at https://www.mdpi.com/article/10.3390/jcm11061617/s1, Table S1: Association of each dietary pattern with the prevalence of early AMD (Category 2), Table S2: Frequency of food/recipe consumption per week according to each dietary pattern, Table S3: Weekly frequency of staple foods consumption at each meal of a day according to each dietary pattern, Table S4: Basic characteristics according to quartile of dietary pattern scores; Table S5: Association of each dietary pattern with the prevalence of intermediate AMD (aged ≥ 50 years).
