# Supplementary material for: Dietary Patterns and Their Associations with Intermediate Age-Related Macular Degeneration in a Japanese Population
Source: J Clin Med. 2022 Mar 15;11(6):1617. doi: 10.3390/jcm11061617 (PMC8955354; doi:10.3390/jcm11061617)
Supplement: Supplementary file 1 [file jcm-11-01617-s001.zip › Table S1.pdf]

Supplemental Table S1. Association of each dietary pattern with the prevalence of early AMD (Category 2).

	Non-case/ Case	Model 1		Model 2	
Dietary pattern	n (%)	Odds Ratio (95% CI)	*p* for trend	Odds Ratio (95% CI)	*p* for trend
Vegetable rich					
Q1	782/44 (5.3)	Reference	0.677	Reference	0.720
Q2	751/68 (8.3)	1.47 (0.98 - 2.20)		1.45 (0.97 - 2.16)	
Q3	740/70 (8.6)	1.44 (0.96 - 2.16)		1.41 (0.94 - 2.12)	
Q4	745/62 (7.7)	1.17 (0.76 - 1.80)		1.16 (0.75 - 1.78)	
Varied staple food					
Q1	727/60 (7.6)	Reference	0.583	Reference	0.608
Q2	755/64 (7.8)	1.02 (0.71 - 1.48)		1.02 (0.71 - 1.48)	
Q3	755/61 (7.5)	0.99 (0.68 - 1.44)		0.98 (0.67 - 1.43)	
Q4	781/59 (7.0)	0.91 (0.62 - 1.33)		0.91 (0.62 - 1.34)	
Animal foods rich					
Q1	739/75 (9.2)	Reference	0.668	Reference	0.711
Q2	738/58 (7.3)	0.86 (0.60 - 1.24)		0.87 (0.61 - 1.26)	
Q3	766/56 (6.8)	0.87 (0.60 - 1.26)		0.88 (0.60 - 1.27)	
Q4	775/55 (6.6)	0.93 (0.63 - 1.36)		0.94 (0.64 - 1.38)	
Seafood rich					
Q1	777/59 (7.1)	Reference	0.324	Reference	0.333
Q2	756/51 (6.3)	0.88 (0.60 - 1.30)		0.87 (0.59 - 1.29)	
Q3	758/63 (7.7)	1.04 (0.71 - 1.53)		1.04 (0.71 - 1.53)	

Q4	727/71 (8.9)	1.15 (0.79 - 1.68)	1.15 (0.79 - 1.67)

Model 1, adjusted for age, sex. Model 2, adjusted for age, sex, smoking status, alcohol consumption, presence of hypertension, diabetes, and dyslipidemia, history of cardiovascular disease. AMD, age-related macular degeneration; sd, standard deviation; CI, confidence interval.
