# Supplementary material for: Dietary Patterns and Their Associations with Intermediate Age-Related Macular Degeneration in a Japanese Population
Source: J Clin Med. 2022 Mar 15;11(6):1617. doi: 10.3390/jcm11061617 (PMC8955354; doi:10.3390/jcm11061617)
Supplement: Supplementary file 1 [file jcm-11-01617-s001.zip › Table S2.pdf]

Supplemental Table S2. Frequency of food/recipe consumption per week according to each dietary pattern

	Vegetable rich					Varied staple food					Animal foods rich					Seafood rich				
	**Q1**	**Q2**	**Q3**	**Q4**	**β coefficient (95%CI) per 1 quartile increment**	**Q1**	**Q2**	**Q3**	**Q4**	**β coefficient (95%CI) per 1 quartile increment**	**Q1**	**Q2**	**Q3**	**Q4**	**β coefficient (95%CI) per 1 quartile increment**	**Q1**	**Q2**	**Q3**	**Q4**	**β coefficient (95%CI) per 1 quartile increment**
**Margarine on bread**	0.8 (1.8)	0.8 (1.8)	0.8 (1.7)	0.9 (1.9)	0.04 (-0.02, 0.09)	0.1 (0.3)	0.3 (0.5)	0.6 (0.9)	2.3 (3.0)	0.67 (0.62, 0.72)	0.6 (1.7)	0.7 (1.6)	0.8 (1.7)	1.1 (2.1)	0.14 (0.09, 0.19)	1.0 (2.0)	0.9 (1.9)	0.8 (1.9)	0.5 (1.4)	-0.13 (-0.18, -0.08)
**Butter on bread**	0.2 (0.7)	0.2 (0.9)	0.2 (0.6)	0.2 (1.0)	0.00 (-0.02, 0.03)	0.1 (0.3)	0.1 (0.3)	0.2 (0.5)	0.5 (1.5)	0.14 (0.12, 0.16)	0.2 (0.9)	0.2 (0.7)	0.2 (0.7)	0.3 (0.9)	0.04 (0.01, 0.06)	0.2 (1.0)	0.2 (0.6)	0.2 (0.8)	0.2 (0.8)	-0.01 (-0.03, 0.02)
**Milk**	3.0 (3.6)	3.5 (3.8)	4.0 (4.0)	4.7 (4.5)	0.55 (0.43, 0.67)	3.0 (3.5)	3.6 (3.9)	3.9 (4.1)	4.7 (4.4)	0.54 (0.42, 0.66)	4.3 (4.2)	3.9 (4.1)	3.6 (4.0)	3.4 (3.8)	-0.32 (-0.44, -0.20)	4.2 (4.4)	3.9 (4.1)	3.6 (3.8)	3.5 (3.9)	-0.24 (-0.36, -0.12)
**Yogurt**	1.5 (2.5)	2.4 (3.0)	2.8 (3.1)	3.7 (4.0)	0.69 (0.59, 0.78)	1.2 (2.0)	2.2 (2.7)	2.7 (3.1)	4.3 (4.1)	0.98 (0.89, 1.07)	3.9 (4.0)	2.5 (3.1)	2.0 (2.5)	1.9 (2.9)	-0.64 (-0.74, -0.55)	2.5 (3.2)	2.6 (3.2)	2.4 (3.1)	2.8 (3.6)	0.07 (-0.02, 0.17)
**Miso soup**	8.1 (6.1)	9.5 (6.0)	10.6 (6.1)	11.2 (6.1)	1.03 (0.85, 1.21)	15.5 (5.7)	9.4 (5.5)	8.2 (5.1)	6.4 (4.2)	-2.84 (-3.00, -2.68)	10.7 (6.0)	9.8 (6.3)	9.5 (6.0)	9.4 (6.3)	-0.42 (-0.60, -0.23)	10.1 (6.3)	9.9 (6.2)	9.5 (6)	10.0 (6.2)	-0.08 (-0.27, 0.11)
**Tofu products**	1.4 (1.7)	2.3 (2.6)	3.1 (3.2)	4.5 (4.0)	1.01 (0.92, 1.10)	3.4 (4.1)	2.5 (2.7)	2.8 (2.9)	2.7 (2.9)	-0.19 (-0.29, -0.10)	3.9 (4.0)	2.4 (2.5)	2.5 (2.7)	2.5 (3.2)	-0.40 (-0.49, -0.30)	2.3 (2.7)	2.4 (2.7)	2.7 (2.8)	4.0 (4.1)	0.53 (0.44, 0.63)
**Soy / Natto (fermented soybeans)**	2.4 (2.4)	3.3 (3.2)	3.9 (3.1)	5.4 (4.0)	0.96 (0.86, 1.06)	4.7 (4.4)	3.8 (3.1)	3.5 (2.9)	3.0 (2.6)	-0.53 (-0.63, -0.43)	4.6 (3.8)	3.6 (3.2)	3.4 (3)	3.5 (3.4)	-0.35 (-0.45, -0.24)	3.5 (3.3)	3.4 (3.1)	3.6 (3.2)	4.5 (3.8)	0.30 (0.20, 0.40)
**Eggs**	2.2 (2.0)	3.0 (2.4)	3.4 (2.3)	4.2 (3.0)	0.62 (0.55, 0.69)	3.7 (3.2)	3.2 (2.4)	3 (2.2)	3.0 (2.4)	-0.22 (-0.30, -0.15)	2.5 (2.0)	2.7 (2.1)	3.3 (2.4)	4.3 (3.1)	0.61 (0.54, 0.69)	3.7 (2.7)	3.2 (2.5)	2.8 (2.2)	3.0 (2.7)	-0.26 (-0.33, -0.18)
**Chicken**	0.9 (0.8)	1.3 (1.1)	1.6 (1.2)	2.2 (2.2)	0.41 (0.36, 0.45)	1.5 (1.6)	1.5 (1.4)	1.5 (1.5)	1.6 (1.6)	0.03 (-0.01, 0.08)	1.1 (1.3)	1.2 (1.1)	1.5 (1.2)	2.2 (2.0)	0.36 (0.31, 0.40)	1.9 (1.9)	1.5 (1.2)	1.3 (1.1)	1.3 (1.5)	-0.20 (-0.25, -0.16)
**Beef / pork**	1.6 (1.2)	2.2 (1.5)	2.8 (1.7)	3.1 (2.4)	0.49 (0.44, 0.54)	2.6 (2.2)	2.4 (1.7)	2.3 (1.6)	2.3 (1.7)	-0.10 (-0.15, -0.05)	1.7 (1.4)	2.0 (1.5)	2.5 (1.5)	3.5 (2.3)	0.57 (0.52, 0.62)	3.4 (2.3)	2.4 (1.4)	2.0 (1.4)	1.8 (1.6)	-0.52 (-0.57, -0.47)
**Liver**	0.2 (0.4)	0.2 (0.4)	0.3 (0.9)	0.3 (0.6)	0.04 (0.02, 0.06)	0.3 (0.9)	0.2 (0.4)	0.2 (0.5)	0.2 (0.4)	-0.02 (-0.04, 0.00)	0.1 (0.3)	0.2 (0.3)	0.2 (0.4)	0.4 (1.0)	0.09 (0.07, 0.11)	0.2 (0.3)	0.2 (0.4)	0.2 (0.5)	0.3 (1.0)	0.06 (0.04, 0.07)
**Processed meat**	1.0 (1.0)	1.5 (1.5)	1.9 (1.7)	2.5 (2.6)	0.49 (0.44, 0.55)	1.7 (1.8)	1.6 (1.5)	1.6 (1.7)	2.0 (2.3)	0.12 (0.06, 0.17)	1.0 (1.4)	1.3 (1.3)	1.8 (1.6)	2.8 (2.4)	0.58 (0.53, 0.64)	2.5 (2.4)	1.7 (1.7)	1.4 (1.4)	1.4 (1.7)	-0.35 (-0.4, -0.29)
**Fish**	3.0 (2.3)	4.0 (2.5)	4.8 (2.7)	6.5 (4.1)	1.13 (1.04, 1.22)	5.5 (4.0)	4.4 (2.9)	4.3 (2.8)	4.1 (2.9)	-0.45 (-0.55, -0.36)	5.6 (3.9)	4.1 (2.7)	4.0 (2.8)	4.5 (3.2)	-0.34 (-0.43, -0.24)	4.4 (3.0)	4.0 (2.7)	4.2 (2.6)	5.7 (4.1)	0.40 (0.30, 0.50)
**Bone-edible small fish**	0.7 (0.9)	1.2 (1.5)	1.6 (1.9)	3.1 (3.3)	0.77 (0.71, 0.83)	2.0 (2.9)	1.5 (1.7)	1.6 (2.2)	1.5 (2.0)	-0.13 (-0.2, -0.07)	2.3 (3.1)	1.4 (1.7)	1.4 (1.9)	1.5 (2.1)	-0.24 (-0.31, -0.17)	1.1 (1.6)	1.2 (1.5)	1.5 (1.8)	2.7 (3.3)	0.52 (0.45, 0.58)
**Canned tuna**	0.3 (0.4)	0.4 (0.5)	0.5 (0.7)	0.8 (1.3)	0.17 (0.15, 0.20)	0.5 (1.0)	0.5 (0.7)	0.5 (0.7)	0.6 (0.9)	0.02 (0.00, 0.05)	0.3 (0.6)	0.4 (0.6)	0.5 (0.7)	0.8 (1.2)	0.15 (0.13, 0.17)	0.5 (0.7)	0.4 (0.6)	0.5 (0.7)	0.6 (1.2)	0.03 (0.01, 0.06)
**Squid / octopus / shrimp / crab**	0.7 (0.6)	1.0 (0.8)	1.2 (1.2)	1.8 (1.9)	0.38 (0.34, 0.41)	1.3 (1.4)	1.1 (1.1)	1.2 (1.3)	1.2 (1.4)	-0.04 (-0.07, 0.00)	0.8 (0.9)	1.0 (0.9)	1.1 (1.1)	1.8 (1.9)	0.32 (0.28, 0.35)	0.9 (0.9)	0.9 (0.8)	1.1 (1.0)	1.8 (2.0)	0.27 (0.23, 0.31)
**Shellfish**	0.5 (0.5)	0.7 (0.7)	0.9 (1.0)	1.5 (1.8)	0.31 (0.27, 0.34)	1.0 (1.2)	0.8 (1.0)	0.9 (1.2)	0.9 (1.3)	-0.02 (-0.05, 0.02)	0.8 (0.9)	0.7 (0.8)	0.9 (1.1)	1.2 (1.7)	0.16 (0.12, 0.19)	0.6 (0.5)	0.6 (0.6)	0.8 (0.8)	1.6 (1.9)	0.32 (0.28, 0.35)
**Fish eggs**	0.5 (0.6)	0.7 (0.9)	0.9 (1.3)	1.1 (1.6)	0.21 (0.17, 0.24)	1.1 (1.6)	0.7 (1.0)	0.8 (1.2)	0.6 (0.8)	-0.15 (-0.18, -0.11)	0.4 (0.6)	0.6 (0.6)	0.8 (0.9)	1.5 (1.9)	0.34 (0.30, 0.37)	0.6 (0.8)	0.7 (0.8)	0.8 (1)	1.2 (1.8)	0.18 (0.14, 0.21)
**Fish paste products**	0.7 (0.7)	1.1 (0.9)	1.5 (1.2)	2.5 (2.4)	0.58 (0.53, 0.62)	1.7 (2.1)	1.4 (1.3)	1.4 (1.5)	1.3 (1.4)	-0.13 (-0.18, -0.08)	1.2 (1.5)	1.1 (1.1)	1.4 (1.5)	2.0 (2.1)	0.26 (0.21, 0.31)	1.7 (2.0)	1.3 (1.2)	1.2 (1.2)	1.5 (1.8)	-0.06 (-0.11, -0.01)
**Tofu products**	1.2 (1.1)	2 (1.5)	2.8 (1.8)	4.2 (2.9)	0.97 (0.91, 1.03)	3.1 (2.8)	2.5 (1.9)	2.3 (2)	2.2 (1.9)	-0.31 (-0.37, -0.24)	2.9 (2.7)	2.2 (1.9)	2.3 (1.9)	2.7 (2.3)	-0.04 (-0.11, 0.02)	3.0 (2.5)	2.4 (2.0)	2.2 (1.9)	2.5 (2.4)	-0.18 (-0.25, -0.12)
**Potatoes**	1.1 (0.9)	1.8 (1.3)	2.7 (1.6)	4.3 (2.8)	1.06 (1.01, 1.11)	2.7 (2.5)	2.4 (1.9)	2.4 (2)	2.4 (2.2)	-0.08 (-0.14, -0.01)	2.9 (2.7)	2.2 (1.8)	2.3 (1.8)	2.5 (2.1)	-0.09 (-0.16, -0.03)	2.8 (2.3)	2.2 (1.9)	2.2 (1.7)	2.7 (2.6)	-0.03 (-0.09, 0.04)
**Pumpkin**	0.5 (0.6)	0.9 (0.8)	1.3 (1.1)	2.5 (2.5)	0.64 (0.59, 0.68)	1.2 (1.7)	1.2 (1.2)	1.4 (1.7)	1.5 (1.9)	0.09 (0.04, 0.14)	1.7 (2.2)	1.2 (1.3)	1.1 (1.2)	1.3 (1.6)	-0.13 (-0.18, -0.08)	1.3 (1.7)	1.2 (1.6)	1.2 (1.3)	1.6 (1.9)	0.11 (0.06, 0.16)
**Carrot**	1.2 (1.0)	2.4 (1.6)	3.4 (1.9)	5.3 (3.2)	1.31 (1.25, 1.37)	3.0 (2.6)	3.0 (2.3)	3.1 (2.7)	3.2 (2.6)	0.10 (0.02, 0.18)	4.0 (3.0)	2.8 (2.3)	2.6 (2.2)	2.9 (2.4)	-0.34 (-0.42, -0.27)	4.0 (3.0)	2.9 (2.2)	2.6 (2.1)	2.8 (2.5)	-0.41 (-0.48, -0.33)
**Broccoli**	0.7 (0.7)	1.3 (1.1)	1.9 (1.5)	3.1 (2.9)	0.76 (0.71, 0.82)	1.6 (1.6)	1.6 (1.7)	1.8 (2.3)	2.0 (2.2)	0.14 (0.09, 0.20)	2.3 (2.5)	1.5 (1.4)	1.5 (1.8)	1.8 (1.9)	-0.15 (-0.20, -0.09)	1.7 (2.0)	1.6 (1.7)	1.6 (1.7)	2.0 (2.3)	0.09 (0.03, 0.14)
**Green leafy vegetables**	1.8 (1.4)	3.5 (2.4)	5.1 (3.2)	7.7 (4.8)	1.95 (1.85, 2.04)	4.8 (4.2)	4.3 (3.7)	4.6 (4.1)	4.3 (3.5)	-0.13 (-0.24, -0.01)	6.8 (5.1)	4.0 (2.9)	3.7 (3.1)	3.6 (3.0)	-1.00 (-1.11, -0.89)	5.7 (4.5)	4.4 (3.8)	3.8 (3.1)	4.2 (3.8)	-0.51 (-0.63, -0.4)
**Other green-yellow vegetables**	1.3 (1.0)	2.4 (1.7)	3.6 (2.4)	5.6 (4.2)	1.41 (1.33, 1.49)	3.1 (3)	3.1 (2.8)	3.3 (3.3)	3.4 (3.1)	0.11 (0.02, 0.21)	4.7 (4.2)	2.8 (2.4)	2.6 (2.1)	2.8 (2.6)	-0.58 (-0.67, -0.49)	4.5 (4.0)	2.8 (2.4)	2.6 (2.3)	2.8 (2.8)	-0.54 (-0.63, -0.45)
**Cabbage**	1.6 (1.1)	2.5 (1.5)	3.8 (2.0)	5.6 (3.6)	1.32 (1.26, 1.39)	3.3 (2.6)	3.1 (2.2)	3.5 (3.1)	3.6 (2.8)	0.13 (0.05, 0.21)	4.6 (3.8)	3 (2.1)	2.9 (2.0)	3.1 (2.2)	-0.45 (-0.53, -0.37)	4.5 (3.4)	3.1 (2.2)	2.9 (2.1)	3.1 (2.6)	-0.45 (-0.53, -0.37)
**Japanese radish**	1.4 (1.1)	2.2 (1.6)	3.3 (1.9)	5.2 (3.4)	1.25 (1.18, 1.31)	3.3 (2.6)	2.9 (2.3)	3 (2.8)	2.9 (2.7)	-0.11 (-0.19, -0.04)	4.0 (3.2)	2.7 (2.2)	2.6 (2.1)	2.8 (2.4)	-0.35 (-0.42, -0.27)	3.4 (2.8)	2.7 (2.2)	2.7 (2.2)	3.3 (3.0)	-0.05 (-0.13, 0.02)
**Dried radish**	0.4 (0.5)	0.5 (0.6)	0.7 (0.8)	1.4 (2.1)	0.33 (0.29, 0.36)	0.7 (1.2)	0.7 (1)	0.8 (1.6)	0.8 (1.1)	0.03 (-0.01, 0.07)	0.9 (1.5)	0.7 (1.2)	0.7 (1.1)	0.8 (1.2)	-0.03 (-0.07, 0.01)	0.6 (0.8)	0.6 (0.7)	0.7 (1.0)	1.2 (2.0)	0.20 (0.16, 0.24)
**Burdock root / bamboo shoot**	0.6 (0.5)	0.9 (0.8)	1.2 (1.0)	2.0 (2.0)	0.45 (0.41, 0.48)	1.2 (1.3)	1.1 (1.1)	1.2 (1.3)	1.2 (1.6)	0.01 (-0.03, 0.05)	1.3 (1.5)	1.1 (1.3)	1.0 (1.0)	1.2 (1.4)	-0.04 (-0.08, 0.00)	1.1 (1.4)	1.0 (1.1)	1.1 (1.1)	1.4 (1.6)	0.11 (0.07, 0.15)
**Other vegetables**	1.9 (1.4)	3.5 (2.1)	4.9 (2.6)	7.2 (4.3)	1.73 (1.65, 1.82)	4.3 (3.3)	4.1 (3.1)	4.5 (3.6)	4.7 (3.6)	0.16 (0.06, 0.26)	5.7 (4.3)	4.0 (2.9)	3.8 (2.8)	4.1 (3.0)	-0.50 (-0.60, -0.40)	6.3 (4.3)	4.1 (2.7)	3.6 (2.6)	3.6 (2.9)	-0.89 (-0.98, -0.79)
**Mushrooms**	1.4 (1.2)	2.5 (1.7)	3.9 (2.0)	5.9 (3.6)	1.48 (1.41, 1.55)	3.2 (2.7)	3.2 (2.4)	3.6 (3.1)	3.7 (3.2)	0.17 (0.09, 0.26)	4.6 (3.4)	2.9 (2.5)	2.9 (2.4)	3.2 (2.7)	-0.41 (-0.49, -0.33)	4.7 (3.5)	3.2 (2.4)	2.8 (2.3)	3.0 (2.6)	-0.57 (-0.65, -0.48)
**Seaweed**	1.2 (1.2)	2.2 (1.9)	3.1 (2.1)	5.3 (3.4)	1.30 (1.23, 1.37)	3.2 (2.8)	2.9 (2.6)	2.8 (2.7)	2.9 (2.8)	-0.10 (-0.19, -0.02)	4.0 (3.3)	2.6 (2.4)	2.5 (2.2)	2.6 (2.5)	-0.42 (-0.5, -0.34)	3.5 (3.0)	2.7 (2.5)	2.6 (2.4)	3.0 (2.9)	-0.14 (-0.22, -0.06)
**Mayonnaise**	1.2 (1.2)	1.7 (1.4)	2.3 (1.8)	2.9 (2.8)	0.57 (0.52, 0.63)	2.1 (2.2)	2 (1.7)	1.9 (2.1)	2 (2)	-0.04 (-0.10, 0.02)	1.3 (1.6)	1.7 (1.8)	2.1 (1.7)	3.0 (2.4)	0.54 (0.48, 0.59)	2.9 (2.6)	2.0 (1.7)	1.7 (1.5)	1.4 (1.7)	-0.48 (-0.54, -0.42)
**Deep-fried food**	1.4 (1.1)	1.8 (1.2)	2.2 (1.5)	2.7 (2.3)	0.42 (0.37, 0.47)	2.4 (2)	2 (1.5)	1.9 (1.6)	1.8 (1.6)	-0.19 (-0.24, -0.14)	1.3 (1.4)	1.7 (1.2)	2.1 (1.3)	3.1 (2.1)	0.59 (0.54, 0.63)	2.7 (2)	2.0 (1.5)	1.8 (1.4)	1.6 (1.6)	-0.35 (-0.40, -0.30)

Supplemental Table S2. Frequency of food/recipe consumption per week according to each dietary pattern (Continued)

	Vegetable rich					Varied staple food					Animal foods rich					Seafood rich				
	**Q1**	**Q2**	**Q3**	**Q4**	**β coefficient (95%CI) per 1 quartile increment**	**Q1**	**Q2**	**Q3**	**Q4**	**β coefficient (95%CI) per 1 quartile increment**	**Q1**	**Q2**	**Q3**	**Q4**	**β coefficient (95%CI) per 1 quartile increment**	**Q1**	**Q2**	**Q3**	**Q4**	**β coefficient (95%CI) per 1 quartile increment**
**Pan-fried food**	1.8 (1.3)	2.8 (1.6)	3.7 (2.1)	5.1 (3.3)	1.06 (0.99, 1.12)	3.5 (2.7)	3.3 (2.3)	3.3 (2.6)	3.2 (2.5)	-0.10 (-0.17, -0.02)	3.2 (2.7)	2.9 (2.3)	3.2 (2.1)	4.2 (2.7)	0.34 (0.26, 0.41)	5.0 (3.2)	3.2 (2.0)	2.7 (1.8)	2.4 (2.0)	-0.83 (-0.90, -0.76)
**Citrus fruits**	1.0 (1.4)	1.7 (1.8)	2.4 (2.4)	3.9 (3.3)	0.93 (0.86, 1.00)	1.6 (1.8)	2 (2.1)	2.6 (2.8)	2.8 (3.2)	0.43 (0.36, 0.51)	3.1 (3.3)	2.1 (2.5)	1.8 (1.8)	1.9 (2.3)	-0.40 (-0.48, -0.32)	2.1 (2.5)	1.9 (2.1)	2.2 (2.4)	2.8 (3.2)	0.26 (0.18, 0.34)
**Other fruits**	1.1 (1.4)	2.1 (2.1)	3.0 (2.4)	4.8 (3.5)	1.19 (1.11, 1.26)	2.1 (2.3)	2.4 (2.4)	3.0 (2.9)	3.5 (3.3)	0.46 (0.37, 0.54)	4.1 (3.5)	2.5 (2.5)	2.2 (2.2)	2.2 (2.4)	-0.61 (-0.69, -0.53)	3.0 (3.2)	2.5 (2.5)	2.6 (2.6)	3.0 (3.0)	0.01 (-0.08, 0.09)
**Peanuts/almonds**	0.4 (0.8)	0.8 (1.4)	0.8 (1.3)	1.5 (2.2)	0.32 (0.28, 0.37)	0.6 (1)	0.7 (1.2)	1.0 (1.7)	1.2 (2)	0.19 (0.14, 0.24)	0.9 (1.7)	0.7 (1.5)	0.8 (1.3)	1.1 (1.7)	0.07 (0.02, 0.12)	0.7 (1.4)	0.7 (1.1)	0.9 (1.4)	1.2 (2.1)	0.17 (0.12, 0.21)
**Western confectionery**	0.5 (0.6)	0.7 (0.9)	0.8 (0.9)	1.1 (1.5)	0.19 (0.16, 0.22)	0.6 (0.8)	0.7 (0.8)	0.8 (0.9)	1.0 (1.5)	0.13 (0.10, 0.16)	0.5 (0.6)	0.6 (0.6)	0.8 (1.1)	1.2 (1.5)	0.24 (0.21, 0.27)	0.9 (1.2)	0.7 (0.9)	0.7 (0.9)	0.7 (1.2)	-0.07 (-0.10, -0.04)
**Japanese confectionery**	0.6 (0.8)	1.0 (1.1)	1.3 (1.4)	2.1 (2.3)	0.48 (0.44, 0.53)	1.0 (1.3)	1.2 (1.4)	1.3 (1.5)	1.6 (2.1)	0.18 (0.13, 0.22)	1.3 (1.7)	1.0 (1.3)	1.2 (1.7)	1.5 (1.8)	0.08 (0.03, 0.12)	1.4 (1.8)	1.2 (1.5)	1.2 (1.5)	1.2 (1.7)	-0.06 (-0.10, -0.01)
**Green tea**	5.9 (6.5)	7.7 (7.3)	9.3 (7.6)	11.7 (7.8)	1.90 (1.69, 2.12)	8.7 (7.7)	8.5 (7.6)	8.8 (7.6)	8.7 (7.5)	0.03 (-0.20, 0.26)	11.5 (8.0)	9.0 (7.7)	7.4 (7.1)	6.8 (6.8)	-1.57 (-1.8, -1.35)	8.1 (7.5)	8.3 (7.5)	8.6 (7.5)	9.7 (7.9)	0.51 (0.29, 0.74)
**Coffee**	7.7 (6.8)	8.8 (6.9)	9.5 (7.0)	9.8 (7.2)	0.70 (0.49, 0.91)	7.8 (6.9)	8.5 (6.8)	8.9 (6.9)	10.5 (7.1)	0.82 (0.62, 1.03)	7.4 (6.7)	8.1 (6.8)	9.5 (7.0)	10.7 (7.2)	1.12 (0.91, 1.32)	12.8 (7.0)	9.9 (6.9)	7.4 (6.2)	5.6 (5.9)	-2.41 (-2.61, -2.22)
**Rice**	15.0 (5.0)	15.8 (4.9)	16.2 (4.4)	16.1 (4.5)	0.36 (0.22, 0.50)	20.0 (1.8)	18.1 (2.8)	14.5 (3.7)	10.6 (3.7)	-3.19 (-3.29, -3.10)	16.2 (4.5)	16.3 (4.6)	15.7 (4.7)	15.0 (5.1)	-0.42 (-0.57, -0.28)	16.3 (4.5)	15.9 (4.7)	15.6 (4.8)	15.4 (4.9)	-0.31 (-0.45, -0.17)
**Bread**	1.7 (2.6)	1.8 (2.5)	1.7 (2.6)	1.9 (2.6)	0.04 (-0.04, 0.12)	0.1 (0.5)	0.5 (0.9)	1.4 (1.8)	5.1 (2.7)	1.57 (1.52, 1.63)	1.9 (2.7)	1.7 (2.6)	1.7 (2.5)	1.8 (2.6)	-0.03 (-0.11, 0.05)	1.9 (2.7)	1.8 (2.6)	1.8 (2.6)	1.6 (2.5)	-0.12 (-0.19, -0.04)
**Noodles**	1.8 (1.9)	1.6 (1.8)	1.7 (1.8)	1.7 (1.8)	-0.02 (-0.08, 0.03)	0.7 (0.9)	1.3 (1.2)	2.2 (1.8)	2.6 (2.3)	0.66 (0.62, 0.71)	1.3 (1.5)	1.5 (1.6)	1.8 (1.8)	2.3 (2.1)	0.32 (0.27, 0.37)	1.6 (1.7)	1.6 (1.7)	1.7 (1.8)	2.0 (1.9)	0.13 (0.08, 0.19)
**Soba-noodles**	0.5 (0.9)	0.4 (0.8)	0.6 (1.1)	0.6 (0.9)	0.06 (0.03, 0.09)	0.2 (0.4)	0.4 (0.5)	0.6 (0.9)	0.9 (1.4)	0.23 (0.2, 0.26)	0.5 (0.9)	0.5 (0.7)	0.5 (0.9)	0.6 (1.1)	0.05 (0.02, 0.08)	0.3 (0.6)	0.4 (0.6)	0.5 (0.9)	0.8 (1.3)	0.16 (0.14, 0.19)

The weekly frequency of foods according to each quartile of each dietary pattern are expressed as mean values (standard deviation).

CI confidence interval.
