# Supplementary material for: Dietary Patterns and Their Associations with Intermediate Age-Related Macular Degeneration in a Japanese Population
Source: J Clin Med. 2022 Mar 15;11(6):1617. doi: 10.3390/jcm11061617 (PMC8955354; doi:10.3390/jcm11061617)
Supplement: Supplementary file 1 [file jcm-11-01617-s001.zip › Table S3.pdf]

**Supplement Table S3. Weekly frequency of staple foods consumption at each meal of a day according to each dietary pattern**

| | Vegetable rich | | | | | Varied staple food | | | | | Animal foods rich | | | | | Seafood rich | | | | |
|---|---|---|---|---|---|---|---|---|---|---|---|---|---|---|---|---|---|---|---|---|
| | Q1 | Q2 | Q3 | Q4 | β coefficient (95%CI) per 1 quartile increment | Q1 | Q2 | Q3 | Q4 | β coefficient (95% CI) per 1 quartile increment | Q1 | Q2 | Q3 | Q4 | β coefficient (95% CI) per 1 quartile increment | Q1 | Q2 | Q3 | Q4 | β coefficient (95% CI) per 1 quartile increment |
| **Rice** | | | | | | | | | | | | | | | | | | | | |
| **At breakfast** | 4.9 (2.9) | 5.4 (2.6) | 5.6 (2.5) | 5.6 (2.5) | 0.20 (0.12, 0.28) | 6.9 (0.6) | 6.6 (1.2) | 5.5 (2.4) | 2.5 (2.9) | -1.43 (-1.49, -1.36) | 5.5 (2.6) | 5.5 (2.6) | 5.5 (2.5) | 5.0 (2.8) | -0.15 (-0.23, -0.07) | 5.3 (2.7) | 5.3 (2.7) | 5.4 (2.6) | 5.6 (2.5) | 0.09 (0.01, 0.17) |
| **At lunch** | 4.7 (2.5) | 4.7 (2.6) | 4.8 (2.5) | 4.7 (2.6) | -0.01 (-0.08, 0.07) | 6.6 (1.0) | 5.7 (1.8) | 3.8 (2.4) | 2.8 (2.6) | -1.32 (-1.38, -1.26) | 4.6 (2.6) | 4.9 (2.5) | 4.7 (2.5) | 4.6 (2.5) | -0.03 (-0.10, 0.05) | 5.1 (2.3) | 4.8 (2.5) | 4.7 (2.5) | 4.3 (2.7) | -0.28 (-0.35, -0.20) |
| **At dinner** | 5.4 (2.3) | 5.7 (2.1) | 5.8 (1.9) | 5.9 (2) | 0.16 (0.10, 0.23) | 6.5 (1.3) | 5.8 (1.9) | 5.2 (2.3) | 5.2 (2.3) | -0.44 (-0.5, -0.38) | 6.0 (1.9) | 5.9 (1.9) | 5.5 (2.2) | 5.4 (2.3) | -0.24 (-0.31, -0.18) | 5.9 (1.8) | 5.8 (2.0) | 5.6 (2.2) | 5.5 (2.2) | -0.12 (-0.19, -0.06) |
| **Bread** | | | | | | | | | | | | | | | | | | | | |
| **At breakfast** | 1.2 (2.3) | 1.1 (2.2) | 1.1 (2.2) | 1.2 (2.2) | -0.03 (-0.10, 0.03) | 0.1 (0.3) | 0.2 (0.5) | 0.7 (1.4) | 3.7 (3.0) | 1.13 (1.07, 1.19) | 1.2 (2.3) | 1.1 (2.2) | 1.1 (2.1) | 1.3 (2.3) | 0.03 (-0.04, 0.10) | 1.3 (2.4) | 1.2 (2.3) | 1.2 (2.2) | 0.9 (2.0) | -0.14 (-0.20, -0.07) |
| **At lunch** | 0.4 (1.2) | 0.6 (1.3) | 0.6 (1.3) | 0.7 (1.4) | 0.07 (0.03, 0.11) | 0.1 (0.3) | 0.2 (0.6) | 0.7 (1.3) | 1.3 (2.0) | 0.41 (0.38, 0.45) | 0.8 (1.6) | 0.5 (1.2) | 0.5 (1.3) | 0.5 (1.1) | -0.08 (-0.12, -0.04) | 0.5 (1.2) | 0.6 (1.5) | 0.6 (1.3) | 0.6 (1.3) | 0.02 (-0.02, 0.05) |
| **At dinner** | 0.1 (0.4) | 0.1 (0.3) | 0.1 (0.3) | 0.1 (0.3) | 0.00 (-0.01, 0.01) | 0 (0.1) | 0 (0.3) | 0.1 (0.3) | 0.1 (0.5) | 0.03 (0.02, 0.04) | 0 (0.3) | 0.1 (0.4) | 0 (0.2) | 0.1 (0.5) | 0.02 (0.01, 0.03) | 0.1 (0.3) | 0 (0.3) | 0.1 (0.4) | 0.1 (0.4) | 0 (-0.01, 0.02) |
| **Noodles** | | | | | | | | | | | | | | | | | | | | |
| **At breakfast** | 0.1 (0.5) | 0.1 (0.5) | 0.1 (0.4) | 0.1 (0.3) | -0.01 (-0.02, 0.00) | 0 (0.2) | 0.1 (0.3) | 0.1 (0.5) | 0.1 (0.6) | 0.03 (0.02, 0.04) | 0.1 (0.2) | 0.1 (0.3) | 0.1 (0.4) | 0.1 (0.6) | 0.03 (0.02, 0.04) | 0.1 (0.4) | 0.1 (0.4) | 0.1 (0.4) | 0.1 (0.4) | 0.01 (0, 0.02) |
| **At lunch** | 1.1 (1.5) | 1.1 (1.5) | 1.1 (1.5) | 1.1 (1.5) | 0.00 (-0.05, 0.04) | 0.4 (0.7) | 0.7 (1) | 1.5 (1.6) | 1.8 (1.9) | 0.53 (0.48, 0.57) | 0.9 (1.4) | 1.0 (1.4) | 1.1 (1.5) | 1.4 (1.6) | 0.16 (0.12, 0.21) | 1.0 (1.4) | 1.0 (1.4) | 1.1 (1.5) | 1.4 (1.7) | 0.13 (0.08, 0.17) |
| **At dinner** | 0.5 (0.9) | 0.4 (0.7) | 0.5 (0.8) | 0.5 (0.8) | -0.01 (-0.03, 0.02) | 0.3 (0.5) | 0.5 (0.6) | 0.5 (0.8) | 0.7 (1.1) | 0.11 (0.09, 0.13) | 0.3 (0.6) | 0.4 (0.7) | 0.5 (0.8) | 0.7 (1.0) | 0.13 (0.11, 0.15) | 0.5 (0.8) | 0.5 (0.7) | 0.5 (0.8) | 0.5 (0.9) | 0.00 (-0.02, 0.02) |
| **Soba-noodles** | | | | | | | | | | | | | | | | | | | | |
| **At breakfast** | 0 (0.1) | 0 (0.3) | 0 (0.2) | 0 (0.2) | 0.00 (0, 0.01) | 0 (0.1) | 0 (0.1) | 0 (0.2) | 0 (0.3) | 0.01 (0, 0.02) | 0 (0.1) | 0 (0.1) | 0 (0.3) | 0 (0.2) | 0.01 (0, 0.01) | 0 (0.1) | 0 (0.1) | 0 (0.1) | 0 (0.3) | 0.01 (0, 0.02) |
| **At lunch** | 0.3 (0.6) | 0.2 (0.6) | 0.3 (0.8) | 0.4 (0.7) | 0.04 (0.02, 0.06) | 0.1 (0.3) | 0.2 (0.4) | 0.4 (0.7) | 0.6 (1.0) | 0.16 (0.14, 0.18) | 0.3 (0.7) | 0.3 (0.6) | 0.3 (0.7) | 0.3 (0.7) | 0.01 (-0.01, 0.03) | 0.2 (0.4) | 0.2 (0.5) | 0.3 (0.7) | 0.5 (0.9) | 0.10 (0.08, 0.12) |
| **At dinner** | 0.2 (0.5) | 0.1 (0.4) | 0.2 (0.5) | 0.2 (0.5) | 0.01 (0, 0.02) | 0.1 (0.3) | 0.2 (0.3) | 0.2 (0.4) | 0.3 (0.7) | 0.06 (0.04, 0.07) | 0.1 (0.4) | 0.2 (0.4) | 0.2 (0.4) | 0.3 (0.6) | 0.04 (0.02, 0.05) | 0.1 (0.3) | 0.1 (0.3) | 0.2 (0.4) | 0.3 (0.7) | 0.05 (0.04, 0.06) |

The weekly frequency of staple foods according to each quartile of each dietary pattern are expressed as mean values (standard deviation).
