# Supplementary material for: Dietary Patterns and Their Associations with Intermediate Age-Related Macular Degeneration in a Japanese Population
Source: J Clin Med. 2022 Mar 15;11(6):1617. doi: 10.3390/jcm11061617 (PMC8955354; doi:10.3390/jcm11061617)
Supplement: Supplementary file 1 [file jcm-11-01617-s001.zip › Table S4.pdf]

Supplemental Table S4. Basic characteristics according to quartile of dietary pattern scores.

	Vegetable rich					Varied staple food					Animal foods rich					Seafood rich				
	**Q1**	**Q2**	**Q3**	**Q4**	**β coefficient (95% CI) per 1 quartile increment**	**Q1**	**Q2**	**Q3**	**Q4**	**β coefficient (95% CI) per 1 quartile increment**	**Q1**	**Q2**	**Q3**	**Q4**	**β coefficient (95% CI) per 1 quartile increment**	**Q1**	**Q2**	**Q3**	**Q4**	**β coefficient (95% CI) per 1 quartile increment**
**Number**	858	858	859	858		858	858	859	858		858	858	859	858		858	858	859	858	
**Men, %**	72.7	46.9	36.0	26.3		60.0	47.7	40.9	33.3		28.1	46.6	50.5	56.6		29.5	45.2	54.0	53.1	
**Age, yo**	60.0 (8.2)	61.3 (8.1)	62.7 (7.3)	64.3 (6.7)	1.46 (1.23, 1.69)	63.0 (7.3)	62.3 (7.5)	61.6 (8.0)	61.4 (8.1)	-0.55 (-0.78, -0.32)	65.1 (6.1)	63.2 (6.6)	61.1 (8.0)	58.9 (8.7)	-2.06 (-2.28, -1.83)	59.9 (8.2)	61.3 (7.7)	62.7 (7.6)	64.4 (6.7)	1.50 (1.28, 1.73)
**Body mass index, kg/m^2**	23.4 (3.3)	23.3 (3.3)	23.3 (3.3)	23.2 (3.1)	-0.08 (-0.18, 0.02)	23.4 (3.3)	23.4 (3.3)	23.3 (3.2)	23.1 (3.1)	-0.12 (-0.22, -0.02)	23.2 (3.3)	23.4 (3.2)	23.2 (3.2)	23.4 (3.3)	0.03 (-0.07, 0.12)	23.2 (3.3)	23.2 (3.3)	23.4 (3.3)	23.4 (3.2)	0.09 (0.00, 0.19)
**Systolic BP, mmHg**	131.3 (19.3)	129.3 (19.2)	130.3 (19.4)	130.4 (19.6)	-0.15 (-0.73, 0.43)	131.1 (18.8)	130.8 (19.2)	130.3 (20.0)	129.1 (19.6)	-0.65 (-1.23, -0.08)	131.1 (19.8)	130.5 (19.3)	129.6 (19.4)	130.1 (19.1)	-0.35 (-0.93, 0.23)	128.3 (19.1)	129.7 (19.5)	131.3 (19.8)	132 (18.9)	1.26 (0.68, 1.83)
**Diastolic BP, mmHg**	78.1 (11.5)	75.8 (11.5)	76.1 (11.8)	74.6 (11.1)	-1.02 (-1.36, -0.68)	76.8 (11.2)	76.2 (11.4)	76.5 (11.9)	75.1 (11.5)	-0.50 (-0.85, -0.16)	75.3 (11.2)	75.8 (11.5)	76.4 (11.2)	77.2 (12.1)	0.62 (0.28, 0.96)	75.0 (11.4)	76.1 (11.6)	76.7 (11.9)	76.9 (11.1)	0.64 (0.30, 0.99)
**Fasting plasma glucose, mg/dL**	103.4 (19.6)	102.2 (18.3)	101.5 (16.3)	100.3 (13.6)	-1.00 (-1.51, -0.49)	102.1 (16.5)	102.7 (19.6)	101.5 (16.5)	101.2 (15.8)	-0.39 (-0.90, 0.12)	101.2 (15.5)	102.5 (17.3)	101.9 (17.2)	101.8 (18.5)	0.14 (-0.37, 0.66)	99.4 (15.7)	100.7 (16.1)	103.4 (18.8)	104 (17.3)	1.65 (1.14, 2.16)
**HemoglobinA1c, %**	5.7 (0.6)	5.7 (0.6)	5.7 (0.6)	5.7 (0.5)	0.00 (-0.02, 0.02)	5.7 (0.6)	5.7 (0.7)	5.7 (0.6)	5.7 (0.5)	-0.01 (-0.03, 0.00)	5.7 (0.5)	5.7 (0.6)	5.7 (0.6)	5.7 (0.6)	-0.02 (-0.04, -0.01)	5.7 (0.6)	5.7 (0.6)	5.7 (0.6)	5.7 (0.6)	0.03 (0.01, 0.05)
**Total cholesterol, mg/dL**	208.6 (34.3)	209.9 (33.5)	210.8 (32.6)	210.7 (33.5)	0.71 (-0.29, 1.71)	205 (33.9)	210.2 (33)	209.6 (32.3)	215.3 (33.9)	3.04 (2.04, 4.04)	211.5 (33.3)	209.2 (33.8)	210.4 (32.8)	209 (34.0)	-0.64 (-1.64, 0.36)	212.2 (33.8)	211.4 (32.6)	209 (34.5)	207.5 (32.8)	-1.68 (-2.68, -0.68)
**HDL cholesterol, mg/dL**	65.5 (17.1)	68.0 (17.1)	68.6 (16.6)	70.2 (16.9)	1.46 (0.96, 1.97)	66.9 (16.7)	68.5 (17.6)	67.5 (16.5)	69.5 (17.1)	0.67 (0.16, 1.18)	69.5 (17.1)	67.2 (16.4)	68.1 (16.9)	67.5 (17.5)	-0.51 (-1.02, 0.00)	69.6 (16.3)	68.7 (17.7)	67.3 (17.1)	66.7 (16.8)	-1.00 (-1.51, -0.50)
**LDL cholesterol, mg/dL**	117.6 (32.1)	120.2 (31.0)	120.9 (29.9)	120.6 (29.5)	0.97 (0.05, 1.89)	115.9 (30.2)	119.5 (30.1)	119.3 (31.1)	124.5 (30.7)	2.55 (1.64, 3.46)	121.0 (29.8)	119.4 (31.8)	120.3 (30.6)	118.6 (30.3)	-0.61 (-1.53, 0.30)	121.8 (30.9)	120.8 (29.4)	118.9 (31.5)	117.8 (30.7)	-1.42 (-2.33, -0.50)
**Triglycerides, mg/dL**	127.6 (101.2)	108.5 (65.2)	106.6 (68.7)	99.5 (54.8)	-8.63 (-10.86, -6.39)	110.7 (68.9)	110.9 (74.2)	114 (94.8)	106.6 (58.2)	-0.91 (-3.16, 1.34)	105.1 (62.1)	113.2 (86.1)	109.5 (67.9)	114.4 (82)	2.42 (0.17, 4.67)	103.8 (62.2)	109.8 (66.3)	113.8 (74.5)	114.9 (93.6)	3.73 (1.48, 5.98)
**Hypertension, %**	49.7	47.4	49.0	46.3		47.7	48.8	49.9	45.9		51.2	49.9	46.6	44.8		42.7	46.2	50.3	53.3	
**Diabetes, %**	13.2	11.0	10.5	10.5		10.5	12.1	12.1	10.4		12.0	12.4	11.2	9.6		7.8	9.2	13.5	14.6	
**Dyslipidemia, %**	52.4	51.3	53.7	52.9		46.3	52.6	54.1	57.3		60.0	53.5	49.4	47.4		51.4	52.3	53.0	53.6	
**Obesity, %**	28.6	27.6	28.2	26.3		27.2	30.4	27.5	25.6		26.0	28.6	28.2	28.0		25.3	28.6	29.1	27.7	
**History of CVD, %**	3.5	4.9	3.5	3.5		3.1	3.8	4.5	3.8		4.3	4.4	2.7	4.0		2.7	3.3	4.0	5.5	
**Smoking status, %**																				
**Never**	35.1	55.7	64.6	74.4		51.2	56.9	59.8	61.9		74.0	58.7	52.0	45.0		66.2	58.7	51.7	53.1	
**Past**	39.3	29.8	25.3	16.3		30.0	27.7	26.8	26.2		20.0	27.5	29.6	33.6		21.4	26.5	31.8	31.0	
**Current**	25.6	14.5	10.1	9.3		18.9	15.4	13.4	11.9		5.9	13.8	18.4	21.4		12.4	14.8	16.5	15.9	
**Current drinker, %**	62.5	49.5	43.7	35.9		55.8	48.6	45.6	41.5		33.0	46.4	54.0	58.2		38.6	47.3	53.3	52.3	
**Alcohol consumption, g/day**	23.3 (29.6)	15.9 (26.5)	12.9 (26.9)	8.5 (18.1)	-4.74 (-5.50, -3.97)	19.4 (26.7)	15.0 (25.3)	15.5 (30.1)	10.7 (21.2)	-2.54 (-3.32, -1.76)	7.7 (18.1)	14.5 (24.5)	17.6 (26.5)	20.9 (32.0)	4.3 (3.53, 5.07)	10.5 (22.0)	14.0 (23.7)	17.9 (27.9)	18.2 (29.8)	2.69 (1.91, 3.46)

The characteristics according to each quartile of each dietary pattern are expressed as mean values (standard deviation).

BP blood pressure, HDL high-density lipoprotein, LDL low-density lipoprotein, CI confidence interval.
