# Supplementary material for: Dietary Patterns and Their Associations with Intermediate Age-Related Macular Degeneration in a Japanese Population
Source: J Clin Med. 2022 Mar 15;11(6):1617. doi: 10.3390/jcm11061617 (PMC8955354; doi:10.3390/jcm11061617)
Supplement: Supplementary file 1 [file jcm-11-01617-s001.zip › Table S5.pdf]

Supplemental Table S5. Association of each dietary pattern with the prevalence of intermediate AMD (aged ≥ 50 years).

	Non-case/Case	Model 1		Model 2	
Dietary pattern	n (%)	Odds Ratio (95% CI)	*p* for trend	Odds Ratio (95% CI)	*p* for trend
Vegetable rich					
Q1	698/ 88 (11.2)	Reference	0.973	Reference	0.988
Q2	679/ 107 (13.6)	1.22 (0.89 - 1.66)		1.21 (0.89 - 1.66)	
Q3	683/ 104 (13.2)	1.11 (0.80 - 1.52)		1.10 (0.80 - 1.52)	
Q4	681/ 105 (13.4)	1.05 (0.75 - 1.45)		1.06 (0.76 - 1.47)	
Varied staple food					
Q1	664/ 122 (15.5)	Reference	0.004	Reference	0.004
Q2	681/ 105 (13.4)	0.88 (0.66 - 1.18)		0.88 (0.66 - 1.17)	
Q3	687/ 100 (12.7)	0.84 (0.63 - 1.12)		0.82 (0.61 - 1.10)	
Q4	709/ 77 (9.8)	0.63 (0.46 - 0.85)		0.63 (0.46 - 0.86)	
Animal foods rich					
Q1	687/ 99 (12.6)	Reference	0.952	Reference	0.496
Q2	675/ 111 (14.1)	1.29 (0.96 - 1.74)		1.29 (0.95 - 1.74)	
Q3	682/ 105 (13.3)	1.30 (0.96 - 1.76)		1.29 (0.95 - 1.75)	
Q4	697/ 89 (11.3)	1.12 (0.81 - 1.54)		1.10 (0.79 - 1.51)	
Seafood rich					
Q1	704/ 82 (10.4)	Reference	0.746	Reference	0.649
Q2	683/ 103 (13.1)	1.20 (0.88 - 1.64)		1.19 (0.87 - 1.63)	
Q3	682/ 105 (13.3)	1.10 (0.8 - 1.51)		1.09 (0.79 - 1.49)	

Q4	672/ 114 (14.5)	1.12 (0.82 - 1.54)	1.10 (0.80 - 1.51)

Model 1, adjusted for age, sex. Model 2, adjusted for age, sex, smoking status, alcohol consumption, presence of hypertension, diabetes, and dyslipidemia, history of cardiovascular disease. AMD, age-related macular degeneration; CI, confidence interval.
